# Supplementary material for: RING domains act as both substrate and enzyme in a catalytic arrangement to drive self-anchored ubiquitination
Source: Nat Commun. 2021 Feb 22;12:1220. doi: 10.1038/s41467-021-21443-6 (PMC7900206; doi:10.1038/s41467-021-21443-6)
Supplement: Supplementary file 1 — Supplementary Information [file 41467_2021_21443_MOESM1_ESM.pdf]

'Supplementary Information

RING domains act as both substrate and enzyme in a catalytic arrangement to drive sel f-anchored ubiquitination

Leo Kiss[1,*], Dean Clift[1], Nadine Renner[1], David Neuhaus[1], Leo C James[1,*]

[1]MRC Laboratory of Molecular Biology
*correspondence: lkiss@mrc-lmb.cam.ac.uk, lcj@mrc-lmb.cam.ac.uk

Content:

Supplementary Figs. 1-13
Supplementary Table 1

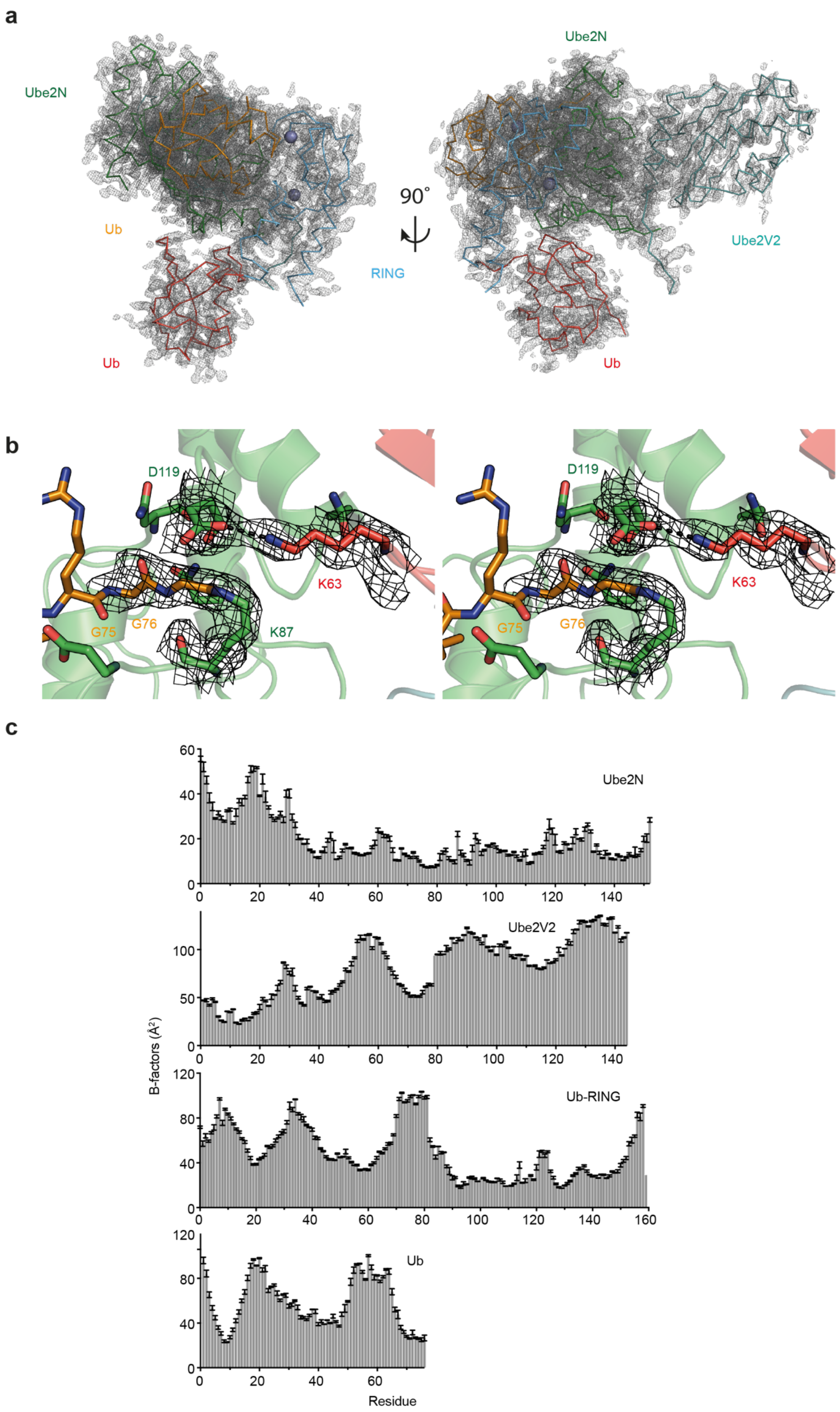

a
Ube2N
Ub
RING
Ub
90˚
Ube2N
Ube2V2
Ub
b
D119
K63
G75
G76
K87
D119
K63
G75
G76
c
Ube2N
Ube2V2
Ub-RING
Ub
B-factors (Å²)
Residue

Supplementary Fig. 1 Crystal structure of Ub-R:Ube2N~Ub:Ube2V2 (7BBD). **a** All proteins contained in the asymmetric unit are shown as ribbon and their $2F_O$-F_C density is shown at 1.0 sigma (Ub-R, Ub in red and R in blue; Ube2N~Ub, Ube2N in green and Ub in orange; Ube2V2 in teal). **b** Stereo image of the active site of Ube2N. $2F_O$-F_C density is shown at 1.0 sigma for selected catalytic residues (Ube2N (green): K87, D119; donor Ubiquitin (orange): G76, G75; acceptor Ubiquitin (red): K63). **c** Shown are B-factors (represented as mean ± standard error of the mean of N, C^{α}, C') for all chains in the Ub-R:Ube2N~Ub:Ube2V2 structure. Ub, ubiquitin; R, RING

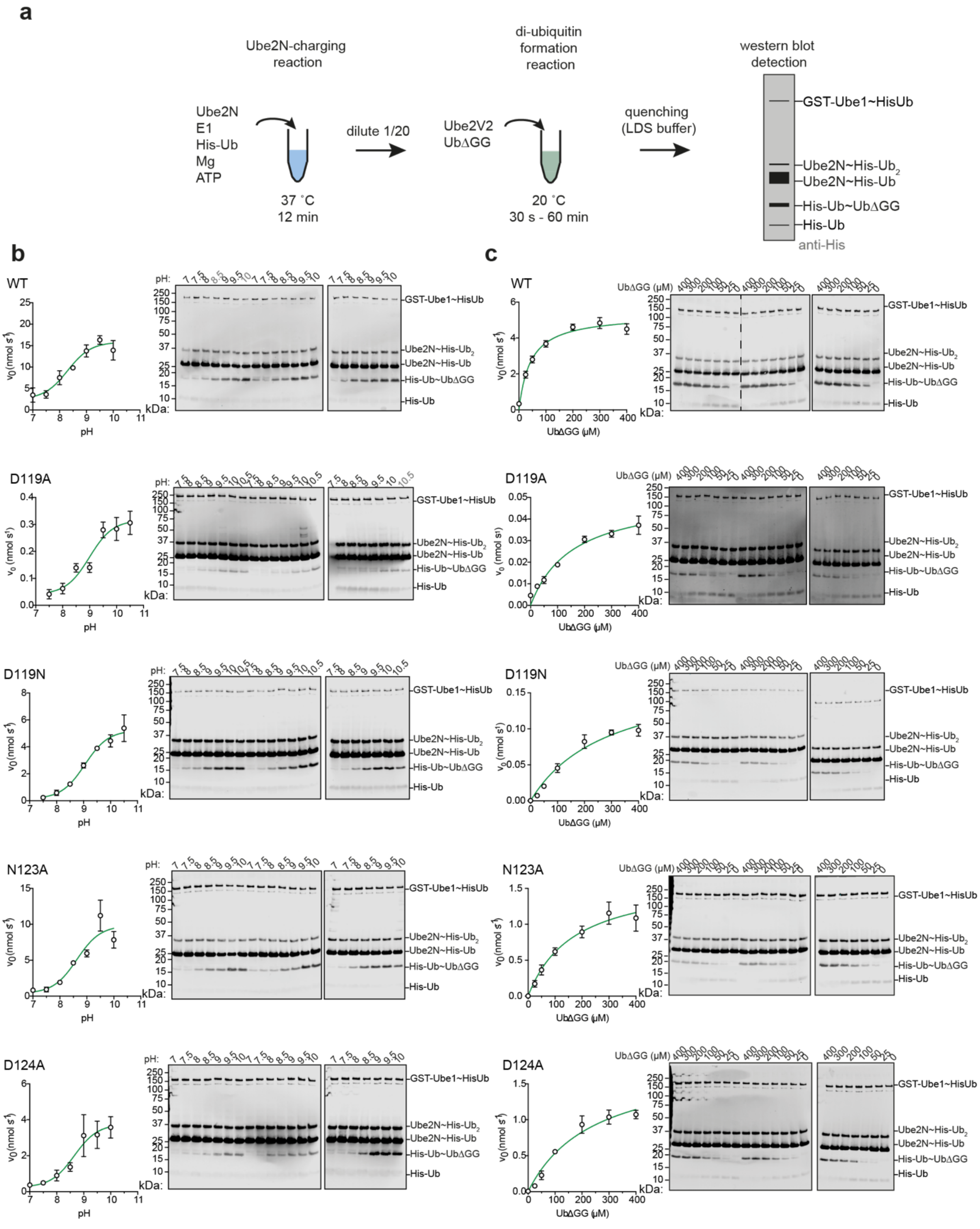

Supplementary Fig. 2 Di-ubiquitination kinetics. **a** Schematic cartoon of Ube2N catalyzed di-ubiquitination kinetic experiments. **b** Plots of pH dependency of kinetics and **c** Michaelis-Menten kinetics and corresponding western blots. Single measurements had to be excluded when one of the bands contained a saturated spot, making quantification impossible. These measurements have the pH/UbΔGG label in gray. Data are presented as mean ± standard error of n = 3 technical replicates. Raw data is provided in Source Data. Ub, ubiquitin; kDa, kilo Dalton

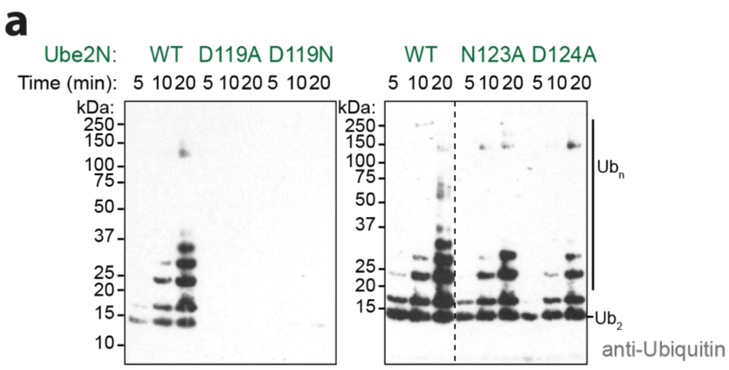

Supplementary Fig. 3 Free ubiquitin chain formation of Ube2N mutants. **a** Western blot of a free ubiquitin chain formation assay using 1 μM TRIM21 RING. Western blot is representative of n = 2 independently performed experiments. **b** Shown is a Coomassie gel of the Ube2N assay stocks. This gel was run once. Uncropped blots and gels are provided in Source Data. Ub, ubiquitin; kDa, kilo Dalton

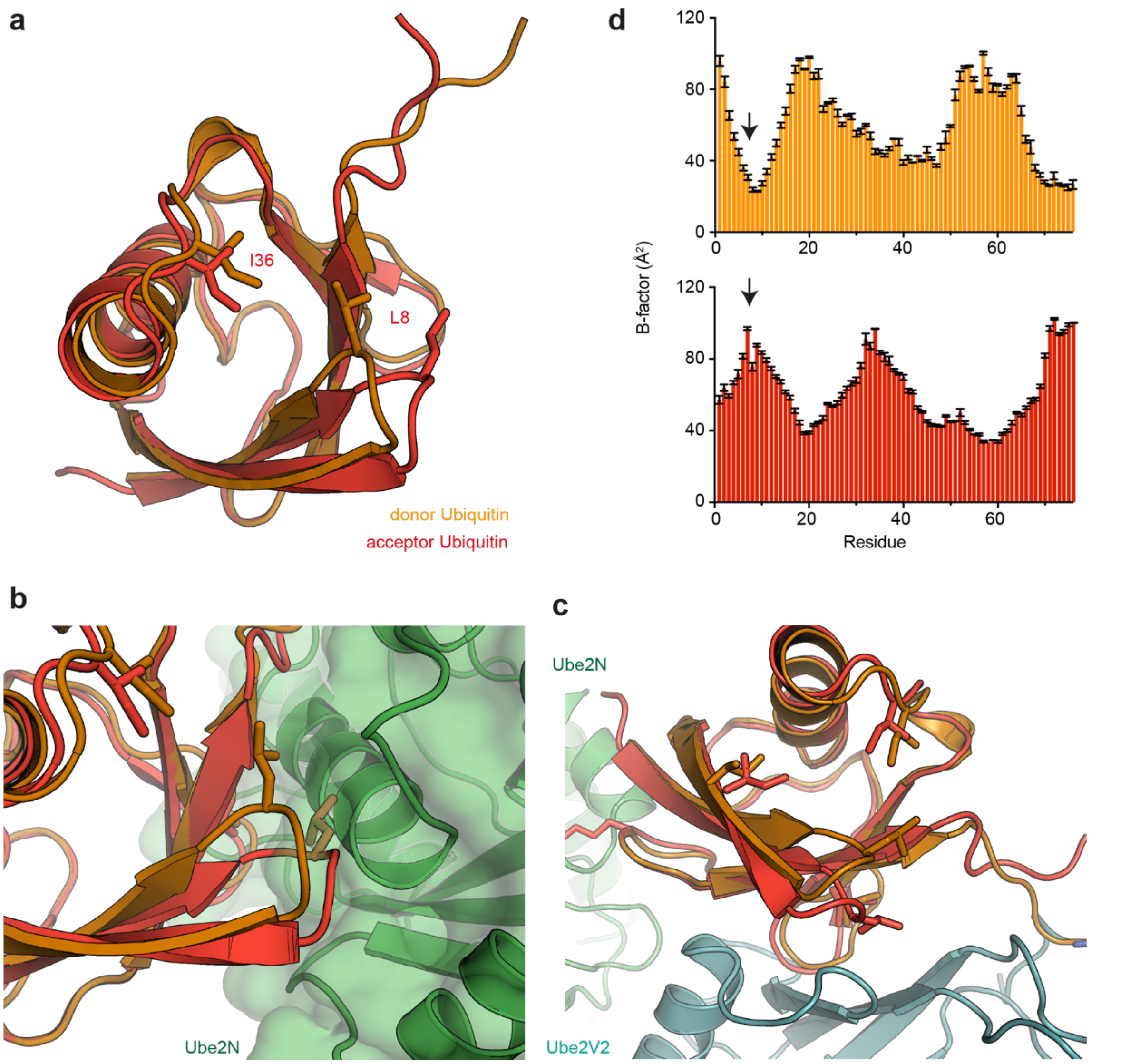

Supplementary Fig. 4 Dynamic ubiquitin loop configurations. **a**, **b**, **c** Structural alignments of the donor (orange) and acceptor ubiquitin (red) found in the Ub-R:Ube2N~Ub:Ube2V2 structure (7BBD, Ube2N in green and Ube2V2 in teal). Main differences can be seen in the β_1-β_2 loop carrying L8, which is either in the loop in (donor ubiquitin, orange) or loop out configuration (acceptor ubiquitin, red). **d** Shown are B-factors (represented as mean ± standard error of the mean of N, C$^{\alpha}$, C' of the Ub-R:Ube2N~Ub:Ube2V2 structure) for the donor and acceptor ubiquitin. Ub, ubiquitin; R, RING

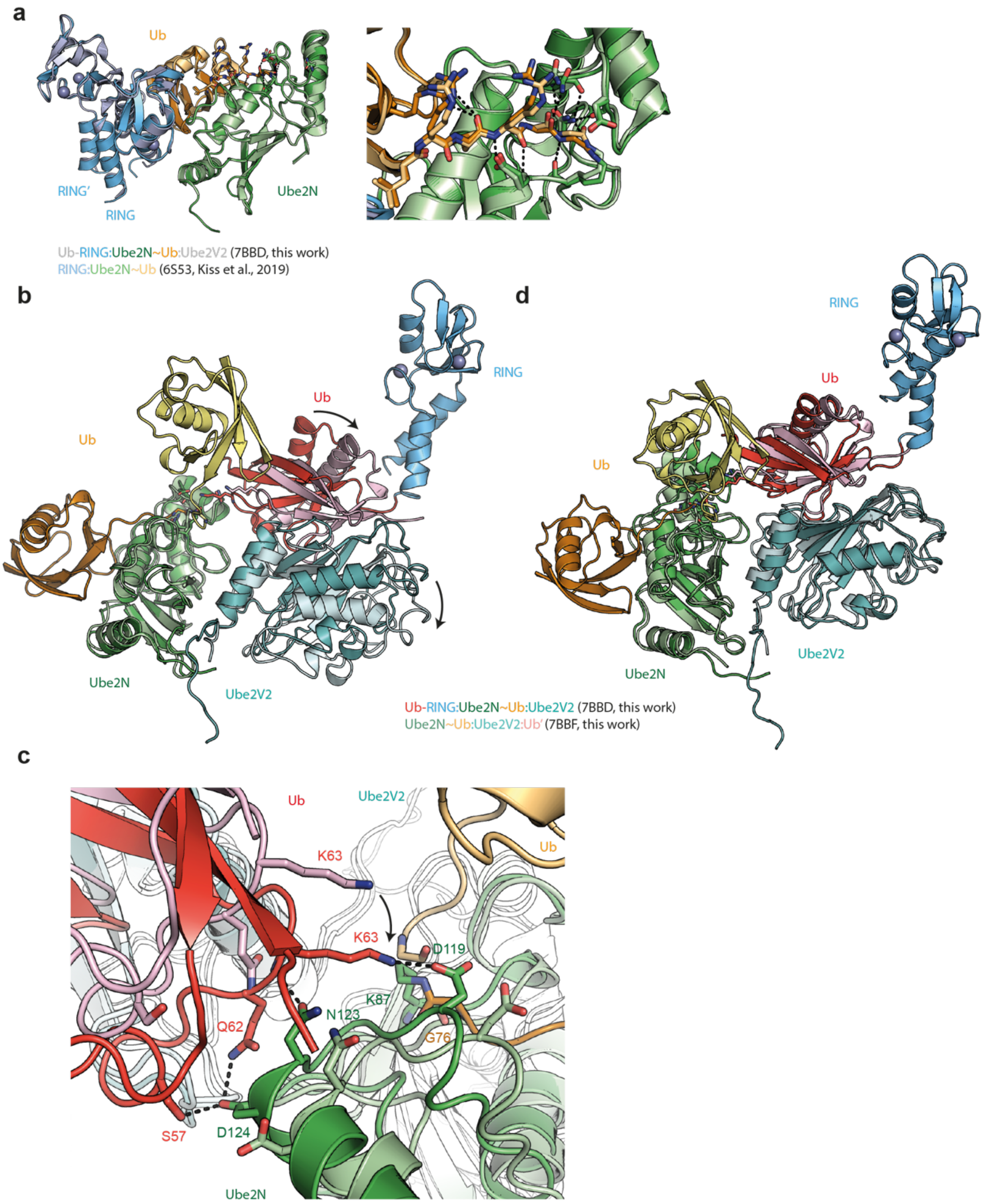

Supplementary Fig. 5 Structural Alignments. **a** Structural alignment between R:Ube2N~Ub (6S53[1]) and Ub-R:Ube2N~Ub:Ube2V2 (7BBD, Ub-R, Ub in red and R in blue; Ube2N~Ub, Ube2N in green and Ub in orange; Ube2V2 in teal). **b** Structural alignment between Ub-R:Ube2N~Ub:Ube2V2 and Ube2N~Ub:Ube2V2 (7BBF). Alignment was performed on Ube2N/Ube2V2. **c** Close up of the alignment shown in **b**. Highlighted are interactions that are different between the two structures and that result in different orientation of the acceptor ubiquitin (red). **d** Structural alignment between Ub-R:Ube2N~Ub:Ube2V2 and Ube2N~Ub:Ube2V2. Alignment was performed on Ube2N~Ub/Ube2V2. Ub, ubiquitin; R, RING

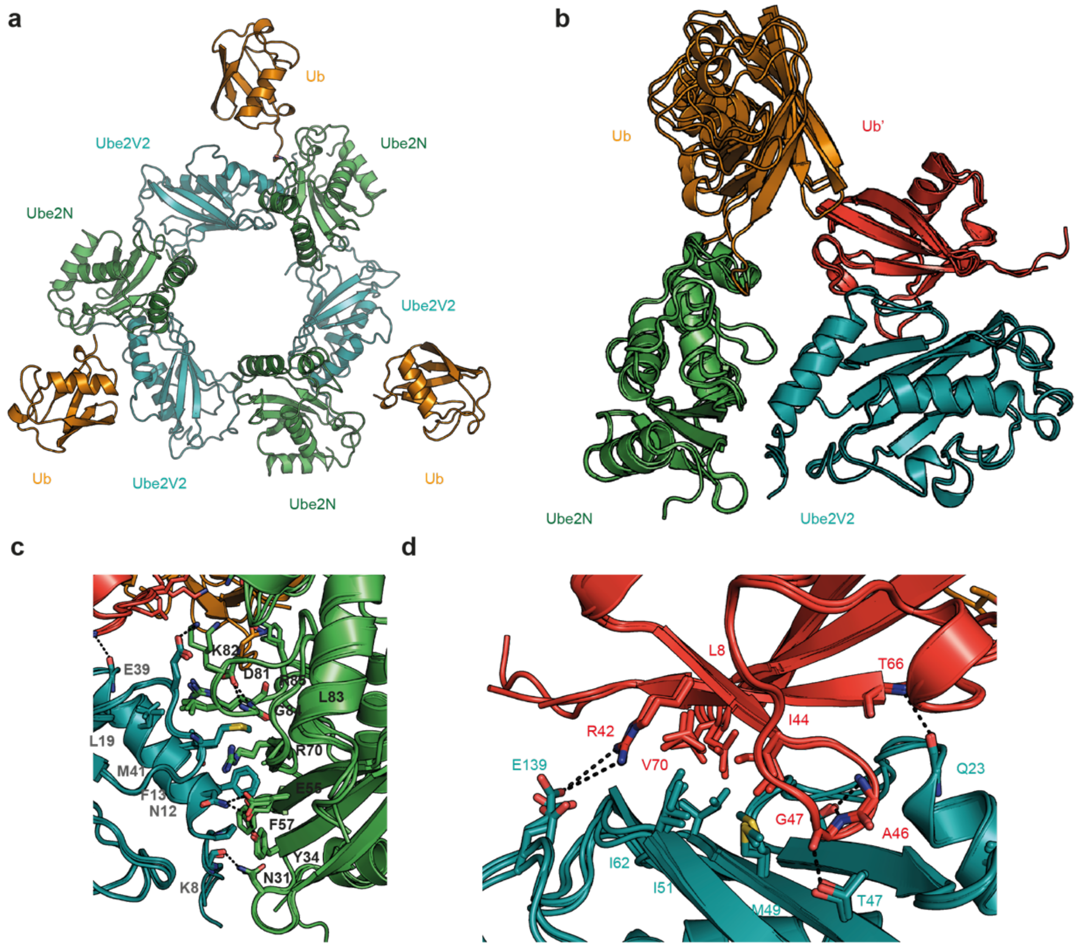

Supplementary Fig. 6 Crystal structure of Ube2N~Ub:UbeV2 (7BBF). **a** Asymmetric unit of Ube2N~Ub:Ube2V2 (Ube2N, green; Ub, orange; Ube2V2, teal) at 2.5 Å resolution. The three copies of Ube2N~Ub:Ube2V2 are related by translational non-crystallographic symmetry. **b** Overlay of the three copies of Ube2N~Ub:Ube2V2. The acceptor ubiquitin (red) was generated by invoking crystal symmetry. **c** Close up of the Ube2N:Ube2V2 binding interface. **d** Close up of the acceptor Ub:Ube2V2 interface. Ub, ubiquitin

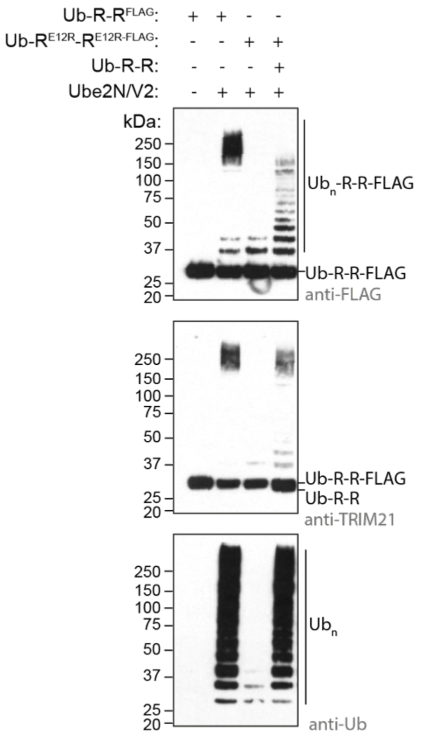

Supplementary Fig. 7 TRIM21-anchored ubiquitination can occur in *trans*. Ubiquitination was incubated for 20 min and performed as other ubiquitination assays but with 50 µM ubiquitin. All TRIM21 constructs are obligate dimers (R-R-fusions) and either tag-free or FLAG-tagged. Ubiquitination deficient Ub-R^{E12R}-R^{E12R}-FLAG can be ubiquitinated in *trans* in presence of FLAG-tag-free Ub-R-R. Western blots are representative of n = 2 independent experiments. Uncropped blots are provided in Source Data. Ub, ubiquitin; R, RING; kDa, kilo Dalton

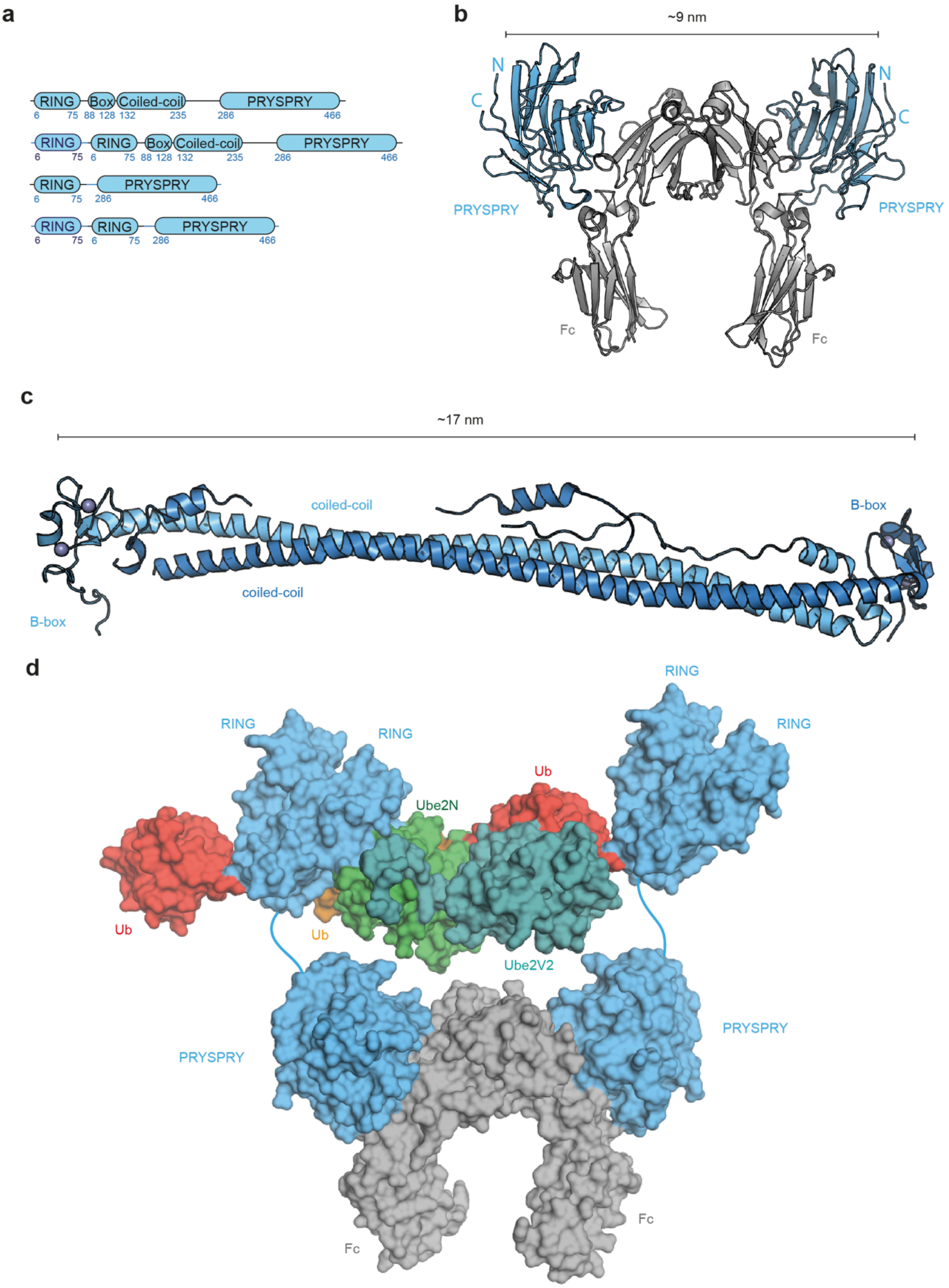

Supplementary Fig. 8 Structural models for distances of different TRIM21 constructs. **a** Domain architecture of TRIM21 constructs used in biochemical and cellular assays. For biochemical experiments, the N-terminus of TRIM21 was mono-ubiquitinated. **b** Structure of TRIM21 PRYSPRY (blue) in complex with Fc (gray, 2IWG[2]). The distance shown spans from

the N-terminal His of one to the other. **c** Structure of TRIM5α-B-Box-coiled-coil (blue, 4TN3[3]). TRIM21 and TRIM5α coiled-coils align well by sequence and show no insertions. Thus, TRIM5α-coiled-coil is a suitable model for the corresponding region of TRIM21. The distance shown spans from the N-terminus of one B-box to the other. **d** Structural model of Ub-R-R-PRYSPRY:Fc during initiation of ubiquitin chain elongation. Our Ub-R:Ube2N~Ub:Ube2V2 (7BBD, Ub-R, Ub in red and R in blue; Ube2N~Ub, Ube2N in green and Ub in orange; Ube2V2 in teal) structure (as the canonical model) was superposed on the TRIM21-PRYSPRY:Fc structure. Lines indicate the linkers between RING and PRYSPRY. Ub, ubiquitin; R, RING

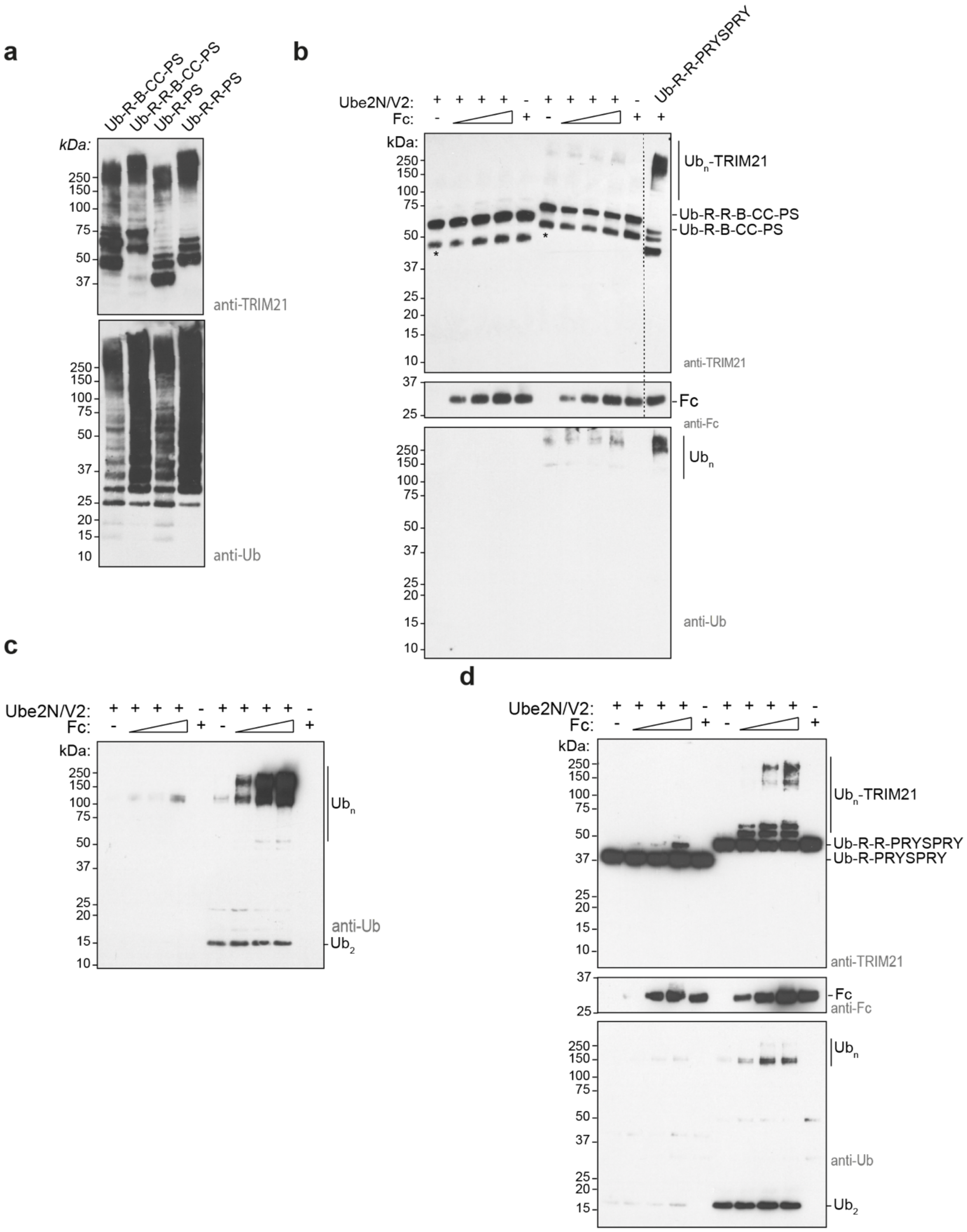

Supplementary Fig. 9 Substrate binding induces catalytic RING topology. **a** Ubiquitin chain formation assay of Ub-TRIM21 constructs after 40 min. Western blot is a representative of n = 2 independent experiments. **b** Substrate (Fc) induced self-ubiquitination assay of 100 nM Ub-TRIM21 constructs. Full blots and additional ubiquitin blot are shown for the data shown in Fig. 3d. For the blot with full-length TRIM21 constructs, Ub-R-R-PS with Fc was also performed as a positive control (dashed line indicates cropping in Fig. 3b). **c** Ubiquitin western blot for the assay shown in Fig. 3d for Ub-R-PS and Ub-R-R-PS. **d** Substrate (Fc) induced self-ubiquitination assay of 50 nM Ub-TRIM21 constructs. Reactions were incubated for 5 min.

*(asterisk) indicates a TRIM21 degradation product that could not be removed during purification. Biochemical assays in **b**, **c**, **d** were performed in n = 3 independent experiments. Uncropped western blots are provided in Source Data. Ub, ubiquitin; R, RING; B, Box; CC, coiled-coil; PS, PRYSPRY; kDa, kilo Dalton

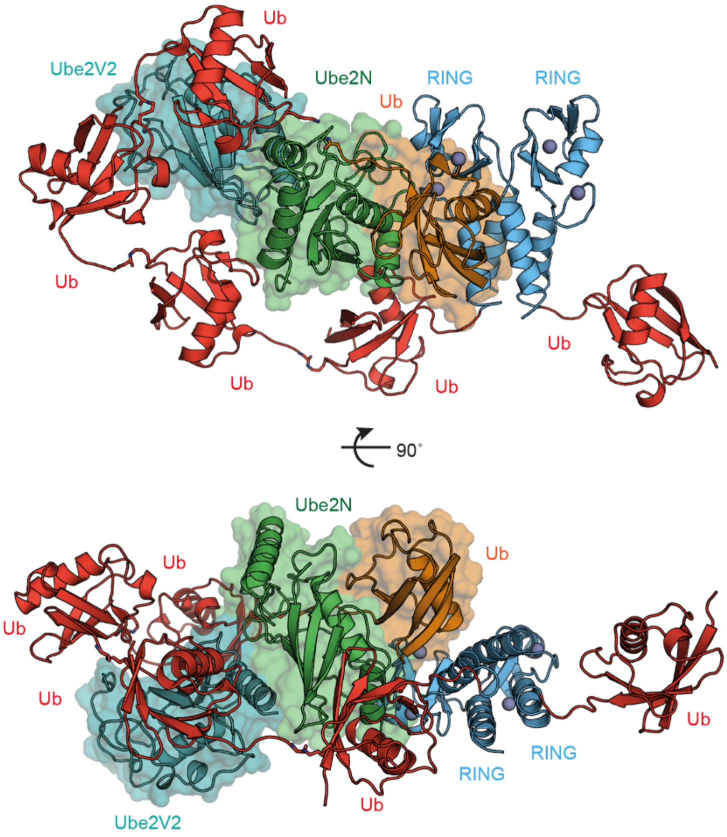

Supplementary Fig. 10 Structural modelling of a *cis*-ubiquitinating TRIM21. In order to achieve ubiquitination in *cis*, the RING-anchored (blue) ubiquitin (red) chain must be sufficiently long to reach the active site on Ube2N~Ub/Ube2V2 (Ube2N in green, Ub in orange, Ube2V2 in teal). The chain can go around two different routes and both cases were modelled in this study. The ubiquitin chain was modelled using the Ub-R:Ube2N~Ub:Ube2V2 structure (7BBD) and a structure of K63-linked Ub_2 (2JF5[4]) using PyMol. For both cases the acceptor ubiquitin was used as orientation for the chain direction. In Fig. 4a the priming (RING-bound) ubiquitin was moved, whereas here it was not. The two central ubiquitin molecules (i.e. numbers 2 and 3) where added by modelling. A RING-anchored ubiquitin chain length of 4 was the shortest found to be possible. Ub, ubiquitin; R, RING

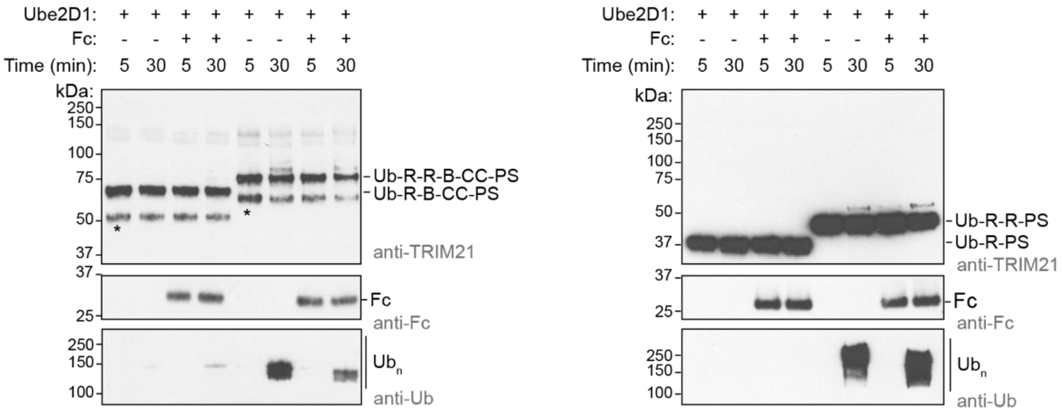

Supplementary Fig. 11 Ube2D1 cannot mediate TRIM21 ubiquitination via the catalytic RING topology. Fc-induced self-ubiquitination assay of 100 nM Ub-TRIM21 in the presence of 0.5 μM Ube2D1. Western blots represent n = 2 independently performed experiments. Uncropped western blots are provided in Source Data. Ub, ubiquitin; R, RING; B, Box; CC, coiled-coil; PS, PRYSPRY; kDa, kilo Dalton

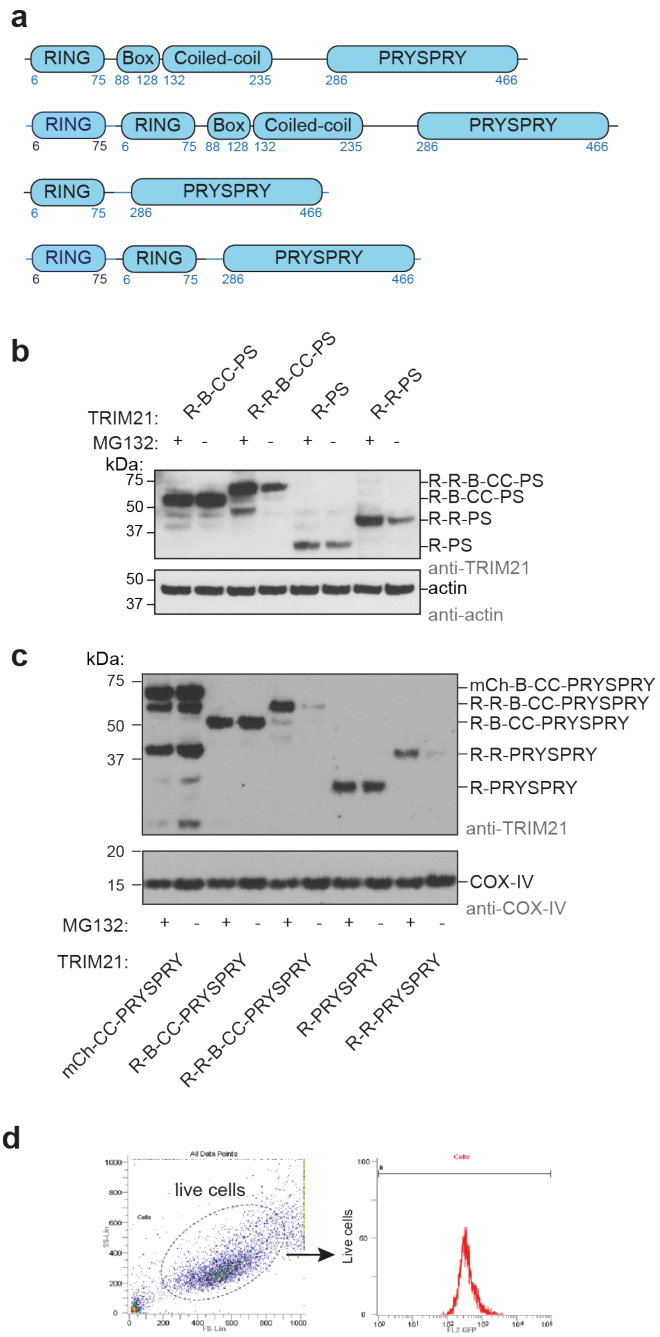

Supplementary Fig. 12 Cell biological analysis of TRIM21 constructs. **a** Domain architecture of TRIM21 constructs used in cellular assays. **b** Transient expression of TRIM21 constructs in *TRIM21*-knock-out RPE1 cells. After electroporation, cells were either treated with MG132 or DMSO. Constructs with constitutive RING dimers show proteasomal turnover. Western blots are representative of n = 2 independent experiments. **c** Exemplary western blots of mEGFP-Fc degradation experiment shown in Fig. 5c. Western blots are representative of n = 3 or more independent experiments. Cells were either treated with MG132 or DMSO. **d** Example for flow cytometry. Cells were measured using forward and side scattering to assess live cells. In addition, green fluorescence was measured. Live cells were selected based on forward and side scattering and only the median GFP fluorescence of live cells was used for further analysis. Uncropped blots are provided in Source Data. R, RING; B, B-box; CC, coiled-coil; PS,

PRYSPRY; kDa, kilo Dalton; SS-Lin, side scattering; FS-Lin, front scattering; FL2-GFP, green fluorescence

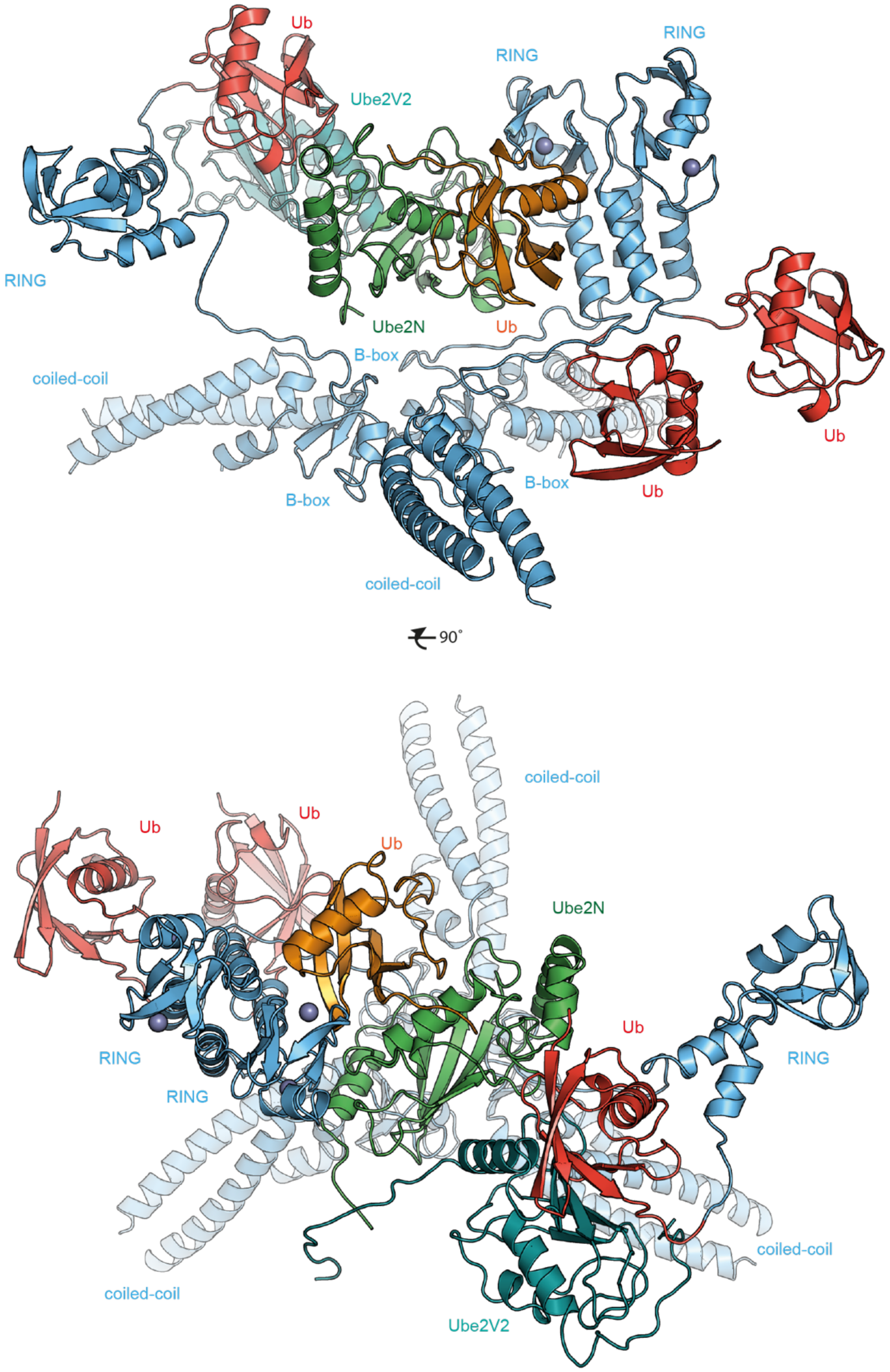
Ub
RING
RING
Ube2V2
RING
Ube2N
Ub
B-box
coiled-coil
Ub
B-box
B-box
Ub
coiled-coil
90°
coiled-coil
Ub
Ub
Ub
Ube2N
Ub
RING
RING
RING
coiled-coil
coiled-coil
Ube2V2

Supplementary Fig. 13 Structural model of catalytic RING topology with TRIM5α. TRIM5 catalytic RING topology model was build based on a TRIM5 trimeric B-CC^{truncated} structure (blue, 5IEA[5]) and the Ub-R:Ube2N~Ub:Ube2V2 structure (7BBD, Ub-R, Ub in red and R in blue; Ube2N~Ub, Ube2N in green and Ub in orange; Ube2V2 in teal). The model for the catalytic RING topology was superposed on the B-CC structure. Linkers between the RING domains and B-boxes were built to connect the domains and the linker between the third RING and its Ub was modelled as being flexible, in line with our B-factor analysis (Supplementary Fig. 1c). R, RING; B, B-box; CC, coiled-coil

Supplementary Table 1 Crystallographic data table. Statistics in highest resolution shell are shown in parentheses. Ub, ubiquitin; R, RING

	**Ub-R:Ube2N~Ub:Ube2V2 (7BBD)**	**Ube2N~Ub:Ube2V2 (7BBF)**
**Wavelength**	0.9762	0.9762
**Resolution range**	19.99 - 2.2 (2.279 - 2.2)	47.74 - 2.542 (2.633 - 2.542)
**Space group**	C 1 2 1	P 32
**Unit cell**	99.15 108.36 75.14 90 104.99 90	145.84 145.84 49.23 90 90 120
**Total reflections**	275272 (27761)	204185 (20653)
**Unique reflections**	38727 (3856)	38540 (3845)
**Multiplicity**	7.1 (7.2)	5.3 (5.4)
**Completeness (%)**	99.41 (99.15)	99.89 (100.00)
**Mean I/sigma(I)**	20.18 (2.23)	15.81 (1.17)
**R-merge**	0.04551 (0.9457)	0.05449 (1.347)
**CC1/2**	1 (0.932)	0.999 (0.497)
**Reflections used in refinement**	38720 (3857)	38506 (3845)
**Reflections used for R-free**	2004 (200)	2017 (203)
**R-work**	0.2222 (0.3245)	0.2081 (0.3483)
**R-free**	0.2523 (0.3285)	0.2479 (0.4045)
**CC(work)**	0.854 (0.362)	0.980 (0.643)
**CC(free)**	0.852 (0.279)	0.964 (0.480)
**Number of non-hydrogen atoms**	4434	8612
**macromolecules**	4147	8573
**ligands**	2	
**solvent**	285	39
**Protein residues**	540	1094
**RMS(bonds)**	0.002	0.015
**RMS(angles)**	0.54	1.41
**Ramachandran favored (%)**	96.98	96.75
**Ramachandran allowed (%)**	3.02	2.79
**Ramachandran outliers (%)**	0	0.46
**Rotamer outliers (%)**	0.67	1.51
**Clashscore**	3.38	16.05
**Average B-factor**	52.09	109
**macromolecules**	52.8	109.11
**ligands**	23.61	
**solvent**	42.01	85.16
**Number of TLS groups**	15	58

Supplementary References

1 Kiss, L. *et al.* A tri-ionic anchor mechanism drives Ube2N-specific recruitment and K63-chain ubiquitination in TRIM ligases. *Nat Commun* **10**, 4502, doi:10.1038/s41467-019-12388-y (2019).

2 James, L. C., Keeble, A. H., Khan, Z., Rhodes, D. A. & Trowsdale, J. Structural basis for PRYSPRY-mediated tripartite motif (TRIM) protein function. *Proc Natl Acad Sci U S A* **104**, 6200-6205, doi:10.1073/pnas.0609174104 (2007).

3 Goldstone, D. C. *et al.* Structural studies of postentry restriction factors reveal antiparallel dimers that enable avid binding to the HIV-1 capsid lattice. *Proc Natl Acad Sci U S A* **111**, 9609-9614, doi:10.1073/pnas.1402448111 (2014).

4 Komander, D. *et al.* Molecular discrimination of structurally equivalent Lys 63-linked and linear polyubiquitin chains. *EMBO Rep* **10**, 466-473, doi:10.1038/embor.2009.55 (2009).

5 Wagner, J. M. *et al.* Mechanism of B-box 2 domain-mediated higher-order assembly of the retroviral restriction factor TRIM5alpha. *Elife* **5**, doi:10.7554/eLife.16309 (2016).
