# Supplementary material for: RING domains act as both substrate and enzyme in a catalytic arrangement to drive self-anchored ubiquitination
Source: Nat Commun. 2021 Feb 22;12:1220. doi: 10.1038/s41467-021-21443-6 (PMC7900206; doi:10.1038/s41467-021-21443-6)
Supplement: Supplementary file 3 — Description of Additional Supplementary Files [file 41467_2021_21443_MOESM3_ESM.docx]

File Name: Supplementary Data 1

Description: Primers, Plasmids and expressed constructs used in this study. Given are the sequences of **a** all primers, **b** all expressed protein sequences and **c** all plasmids used in this study.
