# Supplementary material for: RING domains act as both substrate and enzyme in a catalytic arrangement to drive self-anchored ubiquitination
Source: Nat Commun. 2021 Feb 22;12:1220. doi: 10.1038/s41467-021-21443-6 (PMC7900206; doi:10.1038/s41467-021-21443-6)
Supplement: Supplementary file 5 — Reporting Summary [file 41467_2021_21443_MOESM5_ESM.pdf]

nature research

Corresponding author(s): Leo Kiss, Leo C James

Last updated by author(s): Jan 20, 2021

Reporting Summary

Nature Research wishes to improve the reproducibility of the work that we publish. This form provides structure for consistency and transparency in reporting. For further information on Nature Research policies, see our Editorial Policies and the Editorial Policy Checklist.

Statistics

For all statistical analyses, confirm that the following items are present in the figure legend, table legend, main text, or Methods section.

n/a	Confirmed	
☐	☒	The exact sample size (n) for each experimental group/condition, given as a discrete number and unit of measurement
☐	☒	A statement on whether measurements were taken from distinct samples or whether the same sample was measured repeatedly
☐	☒	The statistical test(s) used AND whether they are one- or two-sided<br>*Only common tests should be described solely by name; describe more complex techniques in the Methods section.*
☒	☐	A description of all covariates tested
☒	☐	A description of any assumptions or corrections, such as tests of normality and adjustment for multiple comparisons
☐	☒	A full description of the statistical parameters including central tendency (e.g. means) or other basic estimates (e.g. regression coefficient) AND variation (e.g. standard deviation) or associated estimates of uncertainty (e.g. confidence intervals)
☐	☒	For null hypothesis testing, the test statistic (e.g. F, t, r) with confidence intervals, effect sizes, degrees of freedom and P value noted<br>*Give P values as exact values whenever suitable.*
☒	☐	For Bayesian analysis, information on the choice of priors and Markov chain Monte Carlo settings
☒	☐	For hierarchical and complex designs, identification of the appropriate level for tests and full reporting of outcomes
☒	☐	Estimates of effect sizes (e.g. Cohen's d, Pearson's r), indicating how they were calculated

Our web collection on statistics for biologists contains articles on many of the points above.

Software and code

Policy information about availability of computer code

Data collection	Crystallographic data collection was performed using the setup provided by the Diamond Light Source
Data analysis	For Crystallography: XDS (Version 31. January 2019), Phenix 1.18.2_3874 and 1.14-3260 (in these versions we used the implemented programs: Phaser, Phenix_Refine), Coot 0.8.9 and 0.9; For general data visualization: Graphpad Prism 7.0d, Pymol 1.8.2.3, Adobe Illustrator v24.2.3, Adobe Photoshop CS6 13.0.6 x64; For Western Blots: Image Studio Lite 5.2.5, ImageJ/FIJI 2.0.0-rc-69/1.52p; For FlowCytometry: ec800 v1.3.6 (Eclipse (iCyt) A02-0058 software); For Incucyte: Incucyte S3

For manuscripts utilizing custom algorithms or software that are central to the research but not yet described in published literature, software must be made available to editors and reviewers. We strongly encourage code deposition in a community repository (e.g. GitHub). See the Nature Research guidelines for submitting code & software for further information.

Data

Policy information about availability of data

All manuscripts must include a data availability statement. This statement should provide the following information, where applicable:

- Accession codes, unique identifiers, or web links for publicly available datasets
- A list of figures that have associated raw data
- A description of any restrictions on data availability

The source data for Figs. 2, 3, 4, 5 and Supplementary Figs. 1, 2, 3, 4, 7, 9, 11, 12 are provided as a Source Data file. The crystal structures are deposited in the Protein Data Bank under the accession codes [http://doi.org/10.2210/pdb7BBD/pdb] and [http://doi.org/10.2210/pdb7BBF/pdb]. All other relevant data are available from the corresponding authors upon reasonable request.

Field-specific reporting

Please select the one below that is the best fit for your research. If you are not sure, read the appropriate sections before making your selection.

☒ Life sciences ☐ Behavioural & social sciences ☐ Ecological, evolutionary & environmental sciences

For a reference copy of the document with all sections, see nature.com/documents/nr-reporting-summary-flat.pdf

Life sciences study design

All studies must disclose on these points even when the disclosure is negative.

Sample size	None of the statistical methods was used to predetermine sample size. To ensure data reproducibility, at least two, mostly three independent replicates were performed for each experiment. The number of replicates for each experiments are specified in the corresponding Figure Legends.
Data exclusions	Data exclusion in crystallographic data set was (outer reflection rejection) was carried out automatically as implemented in the program XDS using pre-established criteria. No other data was excluded.
Replication	All attempts at replicates were reproducible. All experiments were at least performed two times independently. Number of replicates are given in Figure legends.
Randomization	Randomization was not relevant to the experiments performed in this study.
Blinding	Blinding was not relevant to the experiments performed in this study.

Reporting for specific materials, systems and methods

We require information from authors about some types of materials, experimental systems and methods used in many studies. Here, indicate whether each material, system or method listed is relevant to your study. If you are not sure if a list item applies to your research, read the appropriate section before selecting a response.

Materials & experimental systems

n/a	Involved in the study	
☐	☒	Antibodies
☐	☒	Eukaryotic cell lines
☒	☐	Palaeontology and archaeology
☒	☐	Animals and other organisms
☒	☐	Human research participants
☒	☐	Clinical data
☒	☐	Dual use research of concern

Methods

n/a	Involved in the study	
☒	☐	ChIP-seq
☐	☒	Flow cytometry
☒	☐	MRI-based neuroimaging

Antibodies

Antibodies used	anti-His antibody (Clontech, 631212, 1:5,000); Fc: goat antihIgG Fc broad 5211-8004 (1:2,000); TRIM21: rabbit anti-TRIM21 D101D (ST#9204) (1:1,000), Vinculin: rabbit anti-Vinculin EPR8185 ab 217171 (1:50,000); Caveolin-1: rabbit anti-Cav1 (BD: 610059; 1:1,000); anti-Ub-HRP Santa Cruz (sc8017-HRP P4D1; 1:5,000), Mouse monoclonal anti-β-actin-HRP (C4), Santa Cruz, Cat#sc-47778 HRP (1:5,000), RRID:AB_2714189, anti-COXIV, LI-COR Biosciences, Cat#926-42214 (1:5,000); Secondary antibodies were anti-mouse-HRP Sigma (A0168; 1:5,000), anti-rabbit-HRP Cell Signaling (7074; 1:5,000), Goat anti-Rabbit IgG (H+L) Cross-Adsorbed Secondary Antibody, HRP-conjugated, Thermo Fisher Scientific, Cat#31462; 1:5,000
Validation	Each antibody has been validated by the vendor.

Eukaryotic cell lines

Policy information about cell lines

Cell line source(s)	ATCC (RPE-1: CRL-4000; NIH-3T3 by Shvets, E., Bitsikas, V., Howard, G., Hansen, C. G. & Nichols, B. J. Dynamic caveolae exclude bulk membrane proteins and are required for sorting of excess glycosphingolipids. Nat Commun 6, 6867, doi:10.1038/ncomms7867 (2015).)
Authentication	Cell lines originated and authenticated by ATCC using their Short Tandem Repeat (STR) Profiling Cell Authentication Service (see https://www.lgcstandards-atcc.org/Services/Testing_Services/Cell_Authentication_Testing_Service.aspx).

Mycoplasma contamination	We routinely screen our cell lines for mycoplasma by qPCR. All cell lines were tested negative for mycoplasma.
Commonly misidentified lines (See ICLAC register)	None.

Flow Cytometry

Plots

Confirm that:

- [x] The axis labels state the marker and fluorochrome used (e.g. CD4-FITC).
- [x] The axis scales are clearly visible. Include numbers along axes only for bottom left plot of group (a 'group' is an analysis of identical markers).
- [x] All plots are contour plots with outliers or pseudocolor plots.
- [x] A numerical value for number of cells or percentage (with statistics) is provided.

Methodology

Sample preparation	As described in Methods under Flow Cytometry. In short: RPE-1 cells were fixed prior to flow cytometry. For this, cells were resuspended in FACS fixative (4 % formaldehyde, 2 mM EDTA in PBS) and incubated at room temperature for 30 min. Afterwards, cells were centrifuged and resuspended in FACS buffer (2 % FBS, 5 mM EDTA in PSB) and stored at 4 ˚C, wrapped in aluminium foil until use.
Instrument	Eclipse (iCyt) A02-0058 as stated in Methods under Flow Cytometry.
Software	ec800 v1.3.6 (iCyt software)
Cell population abundance	Stopping gate at 10,000 cells.
Gating strategy	As described in Methods under Flow Cytometry. In short: Flow cytometry was performed using an Eclipse (iCyt). Cells were measured using forward and side scattering to assess live cells. In addition, green fluorescence was measured. Live cells were selected based on forward and side scattering and only the median GFP fluorescence of live cells was used for further analysis (see Supplementary Fig 12d).

- [x] Tick this box to confirm that a figure exemplifying the gating strategy is provided in the Supplementary Information.
